# Race to address sexual health inequalities among people of Black Caribbean heritage: could co-production lead to more culturally appropriate guidance and practice?

**DOI:** 10.1136/sextrans-2023-055798

**Published:** 2023-05-03

**Authors:** Melvina Woode Owusu, Mar Estupiñán Fdez. de Mesa, Hamish Mohammed, Makeda Gerressu, Gwenda Hughes, Catherine H Mercer

**Affiliations:** 1 Centre for Population Research in Sexual Health and HIV, University College London, London, UK; 2 School of Health Sciences, Faculty of Health and Medical Sciences, University of Surrey, Guildford, UK; 3 Blood Safety, Hepatitis, STIs and HIV Division, UK Health Security Agency, London, UK; 4 Centre for Population Research in Sexual Health and HIV, Department of Epidemiology and Public Health, University College London Research, London, UK; 5 Department of Infectious Disease Epidemiology and Public Health, London School of Hygiene and Tropical Medicine, London, UK

People of Black Caribbean heritage in the UK experience a disproportionate burden of STIs. This has persisted for decades. Notably, rates of diagnosed chlamydia and gonorrhoea in England among those of Black Caribbean ethnicity were over five times and nearly six times higher, respectively, compared with rates among White ethnic groups, in 2021.^1^ Such health inequalities are widely recognised among racially minoritised communities^2^; for example, the highest COVID-19 mortality rates were observed among non-White racial groups.^3^ Generally, poverty contributes to poorer health outcomes,^2^ and people of Black Caribbean heritage are among the ethnic groups most likely to live in the most socioeconomically deprived areas of England. Even when controlling for deprivation, Black Caribbeans still experience disproportionately high STI diagnosis rates compared with other ethnic groups.^1^ Research by, with and for these communities can help our sector take more informed action to address disparities.

## From research to practice

Research conducted by the UK Health Security Agency and University College London through the National Institute for Health and Care Research (NIHR) Health Protection Research Unit (HPRU) in bloodborne and STIs found that the higher STI diagnosis rates among Black Caribbeans were not attributable to specific clinical, attitudinal or behavioural factors.^4^ Rather, these disparities are likely shaped by the complex interactions between emotional/psychological and interpersonal factors, such as partner concurrency and sociocultural and structural contexts.^5 6^ From this research, three priorities were proposed to help improve individual knowledge and skills which (somewhat) inform individual decision making, for example, improving knowledge of behaviours that increase and, conversely *decrease*, STI risk.^5 6^ The research team considered these draft priorities in the wider context of addressing STI disparities and undertook a co-production exercise. Co-production, as described by the NIHR, requires researchers, practitioners and the public to work together, sharing power and responsibility from the start to the end of the project, including the generation of knowledge.^7^ Applying this co-production approach, our HPRU researchers engaged in two-way knowledge exchange activities alongside stakeholders from, and working with, Black Caribbean communities and together co-produced recommendations to inform culturally appropriate clinical and community-based practices.

## Rationale for co-production

Evidence-based policy and practice are widely supported in health service development and the literature describes the value of involving organisational stakeholders in policy, guidance and service development.^8^ Similarly, patient and public involvement and engagement (PPIE) in research and service development have been increasingly valued over the past decade.^9^ However, PPIE is challenging in the context of sexual health (outside of HIV), reflecting the transient nature of many STIs, a lack of STI-specific ‘service user cohorts’ and continued STI-related stigma.^10^ When STI-related PPIE and stakeholder consultations do occur, activities are often short-term.^9 10^ Further, research teams must navigate challenges, such as the risk of inadvertently perpetuating stereotypes or further marginalising already marginalised communities.

## Co-production in practice: refining draft research priorities and co-producing national guidance and a training resource

The HPRU team identified and worked alongside multiple stakeholder groups regarded as agents of change within the sexual health system. This included service users, community representatives and advocates, service providers, commissioners, health promotion specialists and researchers. All stakeholders stressed the importance of contextualising the research findings within community members’ *lived experiences*. There was consensus about the importance of equipping colleagues with knowledge about inequalities and training in cultural competence to inform and support collaborative programme design and delivery. This resulted in the HPRU research team and stakeholders co-producing and disseminating recommendations and priorities for culturally appropriate guidance and practice:

1. Raise awareness among Black Caribbean communities about STI prevention, transmission risk and diagnosis.
2. Raise awareness among the sector workforce about inequalities in sexual health outcomes disproportionately affecting people of lack Caribbean heritage.
3. Encourage collaborations with local partners and involve Black Caribbean communities.

These recommendations highlight ways in which communities and sexual health provider organisations can take joint ownership of improving the experiences and outcomes for Black Caribbean service users. Since publication of the national guidance^11^ and workforce training video,^12^ public health and sexual health (PHSH) stakeholders, including BASHH’s newly formed Special Interest Group (SIG), the Racially Minoritised Communities SIG have disseminated the outputs, alongside the HPRU team.

Stakeholders reported that co-production helped

1. Raise awareness of STI inequalities and services among community members.
2. Empower and boost capacity among community members and representatives to champion sexual health and well-being among their communities and advocate for better sexual health services.
3. Equip PHSH stakeholders with knowledge to better identify and address Black Caribbean communities’ needs.
4. Establish PHSH champions who took ownership of dissemination, organised video screenings, facilitated group discussions, and achieved buy-in among colleagues.

Importantly, PHSH sector colleagues highlighted that, in addition to their efforts around addressing STI inequalities, there is a broader need for a multifaceted and multiagency response work to address the wider determinants of health, including structural and institutional racism.

## Using co-production in the wider context of equity, equality, diversity and inclusion

Embracing co-production can be facilitated by engaging with the literature on PPIE and considering the current sociopolitical climate. Following the disproportionate impact of the COVID-19 pandemic on racially minoritised communities and the resurgence of the Black Lives Matter movement, we see a renewed focus on the equity, equality, diversity and inclusion agenda and an acceleration towards working with - and for - under-served and often unheard communities in support of social and health justice. This paradigm shift is evidenced within central government through the Department of Health and Social Care’s formation of the Office for Health Improvement and Disparities; among research funders, with the Wellcome Trust’s recent inclusion-focused strategy, NIHR’s formation of the Centre for Dissemination and Engagement and the new Race Equality Framework for Public Involvement in Research. Further, the Lancet has recently established a series on racism, xenophobia, discrimination and health, and networks are forming to support work to address health inequalities including the newly formed Institute of Health Equity Network. Inclusive and collaborative working to address racial and ethnic inequities and disparities in healthcare is increasingly expected and fundamental rather than ‘optional’. The remaining question therefore is ‘how can we work together to meet the needs of groups historically underserved and marginalised?’

We believe that actioning co-production is imperative to inclusive and evidence-based service development and can be facilitated by considering five questions central to knowledge exchange:^7 11^

1. What aspects of (research) knowledge should be communicated and applied?
2. To whom?
3. By whom?
4. How?
5. To what effect or for what purpose?

These critical questions offer a framework (box 1) for research translation and reflective decision making. When considered by multiple stakeholders, this can result in more tailored, targeted delivery of research findings to the end users and beneficiaries of that research. If more widely applied, we believe a co-production approach can provide a mechanism for developing culturally appropriate guidance and practice with and for Black Caribbean communities and other groups that are underserved and address particular disparities as well as inequalities in the wider determinants of health.

Box 1 **Recommendations for using co-production to develop more culturally appropriate guidance and practice.**

**At an early and strategic point (such as at the bid/funding allocation stage),**

- Set the *intention* to apply co-production approaches so that colleagues at all levels understand the rationale for and agree to adopt this approach.
- Assign a budget for co-production.
- Ensure representation and meaningful opportunities for involvement from relevant professional and community stakeholders.

**When identifying and engaging stakeholders,**

- Consider who may be directly or indirectly influenced and/or affected by research and service development.
- Be transparent in your rationale for identifying and engaging stakeholders and acknowledge their expertise.
- Consider how best to establish trust and familiarity among stakeholders, for example, by ensuring visible representation among the team.

**When designing and implementing co-production,**

- Employ the five principles of co-production [7]:
  - Share power with stakeholders.
    The activity is to be jointly owned and include collaboration to achieve a joint understanding.
  - Include all perspectives and skills.
    Make sure the co-production team includes all those who can make a contribution.
  - Respect and value the knowledge of all those working together.
    Everyone is of equal importance.
  - Reciprocate.
    Everybody should benefit from working together.
  - Build and maintain relationships.
    There needs to be joint understanding and consensus and clarity over roles and responsibilities. It is also important to value people.
- Acknowledge that all stakeholders, including community representatives, have conflicting priorities, and so their time should be respected and used wisely (eg, asking them how they would like to be involved and remunerating them for their time).
- Actively promote co-ownership of co-produced resources to support their use ‘on the ground’.
